# Physical Activity, Body Composition and Metabolic Syndrome in Young Adults

**DOI:** 10.1371/journal.pone.0126737

**Published:** 2015-05-20

**Authors:** Minna K. Salonen, Niko Wasenius, Eero Kajantie, Aulikki Lano, Jari Lahti, Kati Heinonen, Katri Räikkönen, Johan G. Eriksson

**Affiliations:** 1 Folkhälsan Research Centre, Helsinki, Finland; 2 Department of Health, National Institute for Health and Welfare, Helsinki, Finland; 3 Department of General Practice and Primary Health Care, University of Helsinki, Helsinki, Finland; 4 Children’s Hospital, Helsinki University Central Hospital, University of Helsinki, Helsinki, Finland; 5 Department of Obstetrics and Gynaecology, MRC Oulu, Oulu University Hospital and University of Oulu, Oulu, Finland; 6 Institute of Behavioural Sciences, University of Helsinki, Helsinki, Finland; Texas A&M University, UNITED STATES

## Abstract

**Objective:**

Low physical activity (PA) is a major risk factor for cardiovascular and metabolic disorders in all age groups. We measured intensity and volume of PA and examined the associations between PA and the metabolic syndrome (MS), its components and body composition among young Finnish adults.

**Research Design and Methods:**

The study comprises 991 men and women born 1985-86, who participated in a clinical study during the years 2009-11 which included assessments of metabolism, body composition and PA. Objectively measured (SenseWear Armband) five-day PA data was available from 737 participants and was expressed in metabolic equivalents of task (MET).

**Results:**

The prevalence of MS ranged between 8-10%. Higher total mean volume (MET-hours) or intensity (MET) were negatively associated with the risk of MS and separate components of MS, while the time spent at sedentary level of PA was positively associated with MS.

**Conclusions:**

MS was prevalent in approximately every tenth of the young adults at the age of 24 years. Higher total mean intensity and volume rates as well as longer duration spent at moderate and vigorous PA level had a beneficial impact on the risk of MS. Longer time spent at the sedentary level of PA increased the risk of MS.

## Introduction

Several non-communicable diseases are strongly linked with overweight and obesity and are affecting a wide range of people in all age groups, including children [1]. At the same time sedentary behavior, e.g. in terms of physical inactivity, has taken over a considerable part of leisure time, particularly in modern societies [2]. In fact, physical inactivity is identified by WHO as the fourth leading risk factor for mortality globally [3].

The metabolic syndrome (MS) is associated with an increased risk for type 2 diabetes and cardiovascular disease. MS is characterized by the co-occurrence of abdominal obesity, dyslipidemia, hyperglycemia and hypertension [4, 5]. The MS is a multifactorial syndrome, which results from the interaction between sedentary behavior, physical inactivity and genetic factors [6]. The prevalence of MS varies according to study population characteristics and definitions applied [7–9]. In the Americas, in Europe, and in India, at least one-fourth of the adult population has the MS [8]. A rather extensive literature exists on the overall prevalence of MS, but only few studies have focused on the prevalence of MS in young adults. A Finnish study reported that the prevalence of MS among 24–39 years old men and women ranged from 9.8% to 14.9% depending on the criteria applied [10].

Regular exercise is associated with several metabolic consequences; exercise reduces visceral fat in adults [11] also without any change in body weight [12], suggesting an increase in muscle mass as a consequence of exercise training. Post-intervention glycated haemoglobin values in patients with type 2 diabetes are significantly lower in physically active groups than in control groups suggesting a reduced risk for type 2 diabetes complications [12]. In addition, regular aerobic exercise has been shown to increase maximal oxygen uptake [13] while resistance training improves glycemic control [14, 15] in type 2 diabetic patients. Benefits of physical activity (PA) on cardiovascular health in terms of lipid and glucose metabolism and inflammation [16] as well as of aerobic training on blood pressure, HDL cholesterol and triglycerides have all been well documented [17–19]. However, studies specifically focusing upon young adults within this area of research are scarce and PA has mostly been assessed by questionnaires.

The aim of this study was to objectively assess measured volume and intensity of total PA and estimate the prevalence of MS in young Finnish adults. We also aimed to study the association between PA and risk of MS, its components and body composition.

## Materials and Methods

### Participants

Arvo Ylppö Longitudinal Study (AYLS) is part of an extensive bi-national multicenter follow-up study conducted in Bavaria, Germany and Uusimaa, Finland [20]. In Uusimaa, Finland, 2193 subjects were recruited from a total of 15311 deliveries, born in one of the seven maternity hospitals between March 15^th^ 1985 and March 14^th^ 1986. Of them 1905 subjects could be traced and they were invited to a follow-up study during the years 2009–2011. Of the invited, 991 subjects, 480 men and 511 women, participated in a clinical study at the Folkhälsan Research Centre, Helsinki, Finland at the mean age of 24.4 (0.6) years.

### Ethics and consent

Approval for the study was obtained from the Coordinating Ethics committee of the Hospital District of Helsinki and Uusimaa. All subjects provided written informed consent before any study procedure was initiated.

### Assessment of anthropometry and cardiometabolic outcomes

Clinical study measurements were performed by a team of trained research nurses. Height was measured without shoes on to the nearest 0.1 cm and weight was measured in light indoor clothing to the nearest 0.1 kg. Body mass index (BMI) was calculated as weight in kilograms divided by the square of height in meters (kg/m^2^). Waist circumference was measured twice midway between the lowest rib and the iliac crest, and the mean of these two measurements was recorded. Body fat percentage was measured by bioelectrical impedance analysis (BIA) using the InBody 3.0 eight-polar tactile electrode system (Biospace Co. Ltd, Seoul, Korea). For three participants, BIA measurements were not applicable.

Plasma glucose concentrations were measured according to the hexokinase method [21]. Serum total cholesterol and triglyceride concentrations were measured with the use of standard enzymatic methods. Blood pressure was measured from the right arm while the subject was in the sitting position and recorded as the mean of two successive readings from a standard sphygmomanometer. Smoking habits were asked from the participants at the clinical visit and educational attainment was obtained from a self-administered questionnaire. MS was defined by using two definitions; the International Diabetes Federation (IDF 2005) criteria [4] and the most recent one, the Joint Interim Statement (JIS) [5]. The IDF criteria include central obesity (waist circumference ≥ 94 cm in men and ≥ 80 cm in women), and at least two of the following factors: serum triglycerides ≥ 1.70 mmol/L or specific treatment for this abnormality; serum HDL cholesterol < 1.03 mmol/L in men and < 1.29 mmol/L in women or specific treatment for this abnormality; systolic blood pressure ≥ 130 mmHg and diastolic blood pressure ≥ 85 mmHg or treatment of previously diagnosed hypertension; fasting plasma glucose ≥ 5.6 mmol/L or previously diagnosed type 2 diabetes. The difference between the IDF and JIS criteria is that the measure for central obesity is a compulsory component in the IDF definition while in the JIS definition three abnormal findings out of the above mentioned 5 would qualify a person for the MS.

### Assessment of PA

In order to obtain objectively measured PA data the subjects were instructed to wear a SenseWear Pro 3 Armband (BodyMedia, Inc., Pittsburg, PA, USA) for 10 consecutive days. The SenseWear armband is a multisensory body monitor, worn on the triceps of the right arm. The monitor enables continuous collection of various physiological and movement parameters through multiple sensors, including a two-axis accelerometer and sensors measuring heat flux, galvanic skin response, skin temperature and near body ambient temperature. Data collected by these sensors are combined with subjects’ characteristics including gender, age and BMI to estimate energy expenditure (volume), intensity of PA and number of steps, using algorithms developed by the manufacturer (SenseWear Professional software, version 6.1). SenseWear Armband has been valid in resting conditions, exercise conditions, and in field monitoring of total daily PA among young adults (on average 20–25 year old) [22–26]. However, it has been found to be less valid in estimating the energy expenditure of high-intensity (> 10 MET) activities or daily energy expenditure of endurance athletes. Despite of these limitations, SenseWear Armband was found to be valid in measuring free-living daily energy expenditure in a general adult population (age range 20–78 years) compared to the double labelled water method. In general, the average risk of error of energy expenditure estimates of accelerometers or similar type of energy expenditure methods have been suggested to be approximately 10% or less [27].

Participants having at least five valid monitoring days, including one weekend day, were included in the analysis. If the participant had more than 5 valid days, the days with the highest amount of data (most measured minutes) were chosen. A valid day consisted of a day with at least 1296 minutes of data, which corresponds to 90% of a whole 24-hour period. To standardize the measurement period to 24 hours across individuals, a 1 metabolic equivalent of task (MET, 1 MET = 3.5 ml O_2_/kg/min or 1 kcal/kg/h), which corresponds to the metabolic rate of sitting at rest, was added for every missing minute [28]. Based on these criteria 737 (75%) out of the 988 men and women, were included in the study.

Minute-by-minute intensity (MET) data of PA produced by the SenseWear armband was divided into sedentary (< 1.5 MET), light (> 1.5–< 3.0 MET), moderate (≥ 3 and < 6 MET) and vigorous (≥ 6 MET) physical activities [29]. For each of these categories duration (hours) of PA were calculated. In addition, duration of combined moderate to vigorous PA was calculated. Total mean intensity (MET) of PA was calculated as the average of SenseWear METs, and total mean volume (MET-hours) was calculated as the average of METs multiplied by the time in hours (MET-hours = MET x duration).

### Statistical analyses

Descriptive results are expressed as mean and standard deviations for continuous variables and percentages for categorical variables, and were calculated separately for men and women. Tests of interaction were conducted to assess whether the effects of PA on MS varied by gender. Since gender specific interactions with PA were not found, further analyses are combined. Linear regression analyses were conducted to explore the associations between total mean intensity (MET-averages) and total mean volume (MET-hours) of PA with body composition and the components of MS. Total mean intensity was standardized to attain more sensible interpretation. For these analyses triglycerides was log-transformed due to skewed distribution. The odds ratios (ORs) and 95% confidence intervals (CI) between the prevalence of MS and total mean intensity (MET) and volume (MET-hours) of PA were calculated with logistic regression analyses. In addition, the ORs were calculated between presence of MS and the time spent (duration) firstly at each level of PA, secondly at moderate to vigorous activity level and thirdly according to three categories of moderate to vigorous activity. The categorization was based on the current physical activity guidelines [30]: <30 min/day, between 30–60 min/day and ≥60 min/day, and the ORs were calculated against the reference group being the least active one. All these models were firstly adjusted for age, sex, smoking and education (model 1). In the second models total mean intensity and volume were further adjusted for BMI including all the previous adjustments (model 2^a^) whereas models for duration being at sedentary or light level were adjusted for duration of moderate to vigorous PA, and models for moderate, vigorous and moderate to vigorous were adjusted for sedentary time (model 2^b^), which were done in order to examine independent effects of sedentary (or less active) and moderate to vigorous PA on MS.

## Results

### Descriptive characteristics and MS

Descriptive characteristics of the study population are shown in Table 1. Approximately every tenth study participant was obese (≥ 30 kg/m^2^). For both genders, around 8% had MS according to the IDF criteria. In women the prevalence of MS was similar when using the JIS criteria, while a slightly higher proportion (10%) of men fulfilled the JIS criteria compared to the IDF criteria. After exclusion of those with incomplete data on PA, prevalence of MS among the 737 participants remained similar; the prevalence of MS according to both MS criteria decreased approximately 1% in both genders. 24% of the men had waist circumference ≥ 94 cm, 23% had elevated blood pressure (≥ 130 mmHg for systolic and ≥ 85 mmHg for diastolic), 9% low HDL cholesterol (< 1.03 mmol/L), 13% high triglyceride (≥1.7 mmol/L) and 12% had high fasting glucose (≥ 5.6 mmol/L) concentrations. Among women, 35% had a waist circumference ≥ 80 cm, 10% had elevated blood pressure, 15% low HDL cholesterol levels (< 1.29 mmol/L), 8% had high triglyceride and 8% had elevated fasting glucose concentrations.

**Table 1:** Characteristics of 988 men and women.

	Men (N = 479)		Women (N = 509)	
	Mean	SD	Mean	SD
Age (years)	24.4	0.6	24.4	0.6
Weight (kg)	80.3	15.2	64.5	13.1
Height (cm)	180.0	6.7	166.1	6.4
BMI (kg/m^2^)	24.8	4.4	23.3	4.5
Lean body mass (kg)	65.2	8.9	45.6	6.0
Percentage of body fat (%)	17.9	6.9	27.3	7.6
Waist circumference (cm)	87.8	11.7	77.9	11.1
Triglycerides (mmol/L)	1.1	0.6	1.1	0.6
HDL cholesterol (mmol/L)	1.33	0.3	1.66	0.4
Systolic blood pressure (mmHg)	138.4	13.1	122.0	11.2
Diastolic blood pressure (mmHg)	78.4	9.0	75.2	8.5
Fasting plasma glucose (mmol/L)	5.2	0.5	5.0	1.0
Obese n (%)	50 (10.4)		47 (9.2)	
Metabolic syndrome (N/%) according to IDF criteria	37 (7.7)		39 (7.7)	
Metabolic syndrome (N/%) according to JIS criteria	46 (9.6)		39 (7.7)	
Smoker (N/%)	133 (36)		101 (27)	
Education (years studied)	14.7	2.4	15.2	2.4

### Physical activity

An average daily wear time of the Armband was 1422 minutes (SD 13.7) for men, 1424 minutes (SD 13.4) for women (23.7 hours for both genders). When using cut off points of < 1.5 MET for sedentary, ≥ 1.5–< 3.0 for light, ≥ 3–< 6 MET for moderate and ≥ 6 MET for vigorous PA, 90% of the men and women spent over 60 minutes per day at least at the level of moderate PA. By applying these thresholds the overall daily pattern of PA (duration, intensity and volume) was similar in men and women. Table 2 shows an average daily duration of PA at each activity level.

**Table 2:** Average daily duration of physical activity among 737 men and women during a five day period.

	Men (N = 365)		Women (N = 372)	
	*Mean	(SD)	*Mean	(SD)
**Average wear time (minutes/day)**	1422	(13.7)	1424	(13.4)
**Sedentary (< 1.5 MET)**				
Duration (hours/day)	17.9	(1.9)	17.3	(2.0)
**Light (**≥ **1.5–< 3.0 MET)**				
Duration (hours/day)	3.4	(1.1)	4.2	(1.3)
**Moderate (**≥**3–< 6 MET)**				
Duration (hours/day)	2.5	(1.5)	2.3	(1.4)
**Vigorous (**≥ **6 MET)**				
Duration (hours/day)	0.5	(0.8)	0.5	(0.6)

*Mean indicates the mean average values for five-day duration of PA; duration indicates average hours / day spent at each intensity level.

### Physical activity and the components of MS

Associations between the components of MS and total mean volume (MET-hours) and intensity (MET) of PA are shown in Table 3. Total mean volume of PA was negatively associated with all components of MS, except for a positive association with HDL cholesterol. Adjustment for total sedentary time did not attenuate these findings. Further adjustment for BMI attenuated some of the results; associations between volume and diastolic blood pressure, triglycerides and fasting glucose were no longer statistically significant. Associations between total mean intensity and the components of MS were found to be quite similar, although after adjustment for total sedentary time, the associations with HDL-cholesterol and fasting glucose became non-significant. All the associations, except for waist circumference, were no longer significant when BMI was taken into account.

**Table 3:** Total mean MET-hours (volume) and intensity (MET) of PA in relation to components of metabolic syndrome.

	Waist circumference		Systolic blood pressure		Diastolic blood pressure		HDL cholesterol		Triglycerides^#^		Fasting glucose	
Volume	β	95% CI	β	95% CI	β	95% CI	β	95% CI	β	95% CI	β	95% CI
Model 1	-1.1***	-1.2, -0.99	-0.5***	-0.6, -0.4	-0.4***	-0.5, -0.3	0.02***	0.01, 0.02	-0.01***	-0.013,- 0.007	-0.01***	-0.02, -0.004
Model 2	-2.6***	-0.03, -0.02	-1.4***	-1.8, -1.0	-0.8***	-1.1, -0.5	0.03***	0.02, 0.04	-0.02***	-0.03, -0.01	-0.03***	-0.05, -0.01
Model 3	-0.3**	-0.5, -0.01	-0.6*	-1.1, -0.1	-0.3^ns^	-0.6, 0.1	0.02**	0.01, 0.03	-0.003^ns^	-0.01, 0.01	-0.01^ns^	-0.03, 0.01
Intensity												
Model 1	-5.3***	-6.0, -4.6	-2.3***	-3.2, -1.4	-1.9***	-2.5, -1.3	0.1***	0.03, 0.07	-0.05***	-0.07, -0.03	-0.05**	-0.09, -0.02
Model 2	-3.6***	-4.5, -2.7	-1.6**	-2.7, -0.5	-1.2**	-2.1, -0.4	0.01^ns^	-0.02, 0.04	-0.03*	-0.05, -0.004	-0.04^ns^	-0.09, 0.001
Model 3	-0.6**	-0.99, -0.16	-0.32^ns^	-1.4, 0.8	-0.5^ns^	-1.4, 0.3	-0.01^ns^	-0.04, 0.02	-0.01^ns^	-0.03, -0.02	-0.01^ns^	-0.06, 0.03

Models 1 are adjusted for age, sex, education and smoking; Models 2 are additionally adjusted for the time spent at sedentary activity level; Models 3, including all the previous adjustments, are also adjusted for BMI ^#^triglycerides are log-transformed due to skewed distribution

*^)^ P < 0.05;

**^)^ P < 0.01;

***^)^ P < 0.001;

^ns)^ Non significant.

### Physical activity, body composition and metabolic syndrome

**Fig 1:**
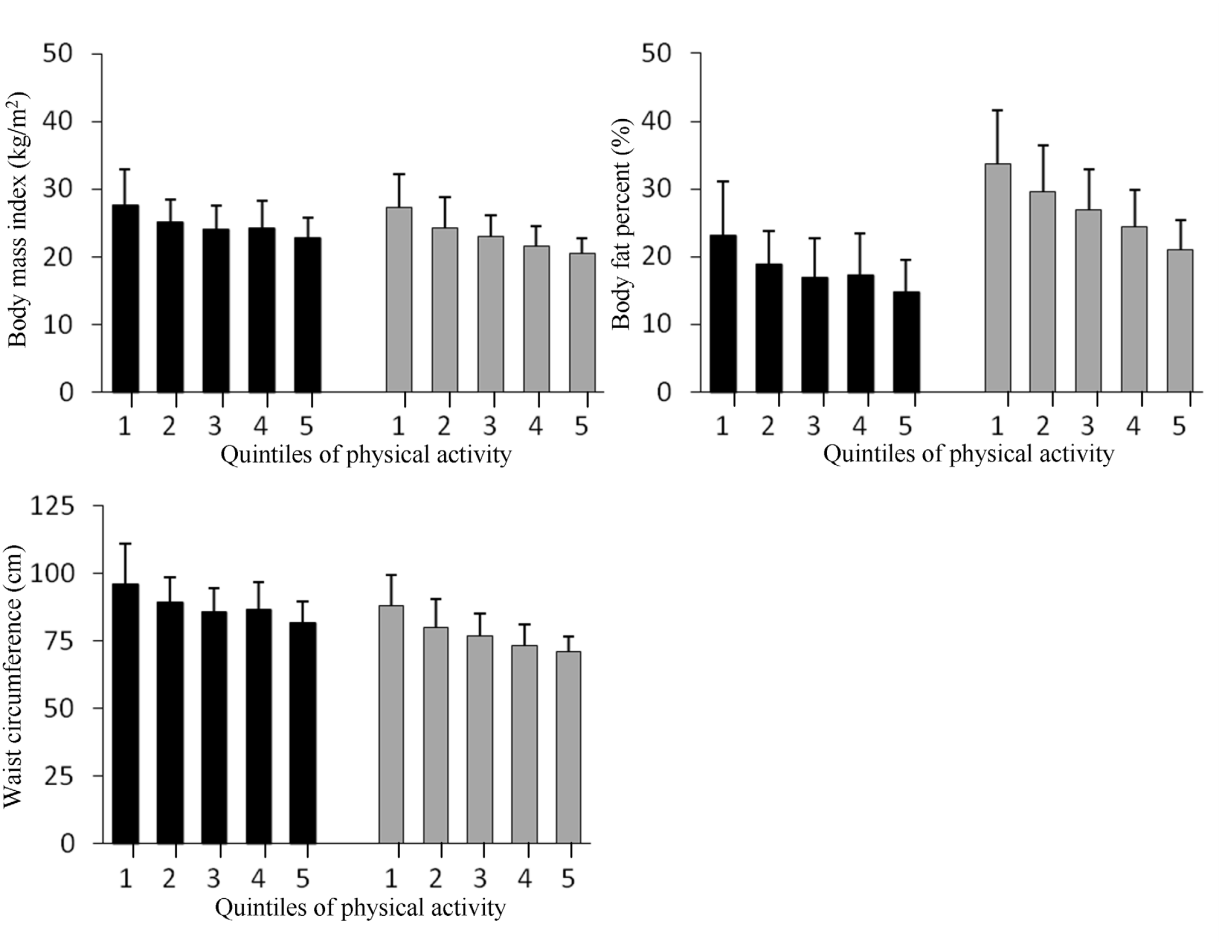
Body composition and physical activity. Mean and standard deviations of body mass index (kg/m^2^), waist circumference and body fat percentage according to physical activity quintiles of total volume (MET-hours) in men (black bars) and women (gray bars). MET-hours ranges for the quintiles were: 19.92–33.22, 33.25–36.39, 36.39–38.87, 38.90–42.77 and 42.79–60.34, respectively. Inverse associations between volume and body composition measures, adjusted for age, smoking and educational attainment were statistically significant in both genders (*P* for trend < 0.001 for all).

Time spent at the levels of sedentary (< 1.5 MET), light (≥ 1.5–< 3.0 MET), moderate (≥ 3- < 6 MET), vigorous (> 6 MET) and moderate to vigorous PA (≥ 3 MET) were examined in association to body composition. Increasing duration of sedentary and light activity, adjusted for age, sex, smoking and educational attainment, was positively associated with BMI, waist circumference and body fat percentage (all p-values < 0.001), while the time spent in moderate and vigorous level PA was negatively associated with all measures of body composition (p < 0.001 for all). Increasing total mean volume (MET-hour) of PA was inversely and significantly associated with all the body composition measures (p < 0.001 for all). The decreasing trends of body composition measures according to increasing volume of total PA (ranked in to quintiles, ranges being 19.51–33.07, 33.08–36.10, 36.11–38.70, 38.70–42.53 and 42.56–60.40, respectively) are shown in the Fig 1.

Odds ratios and 95% confidence intervals for MS (using IDF criteria) according to determinants of PA are shown in Table 4. Odds ratios for MS were calculated according to both, IDF and JIS criteria and the results were similar for both (data not shown). An increase in total mean intensity (MET) and total mean volume (MET-hours) were associated with a decreased likelihood having the MS and remained so after adjustment for BMI. Increasing time spent at sedentary level of intensity was associated with and increased likelihood having the MS, but after adjustment for the time spent at moderate to vigorous level the association attenuated and became non-significant. However, increasing time spent at moderate to vigorous level of PA was associated with lower odds for having the MS, and remained so after adjustment for the time spent at sedentary level of PA. In addition, compared to those engaging less than 30 minutes in moderate to vigorous PA per day, those reaching 30 to 60 minutes of moderate to vigorous PA daily were 72 percent less likely to have MS after adjustments for the confounders used in model 1. Further adjustment for total sedentary time weakened the association. Those who engaged more than 60 minutes in moderate to vigorous PA daily had a very low likelihood to have MS (OR = 0.10, 95% CI: 0.01–0.21) after all the adjustments including total sedentary time.

**Table 4:** ORs and 95% CIs for MS according to IDF criteria by intensity, volume and duration of physical activity.

	Metabolic syndrome according to IDF					
	Model 1			Model 2^a^		
Determinants of physical activity	OR	95% CI	*p*-value	OR	95% CI	*p*-value
Total mean intensity (MET)	0.12	0.06–0.24	< 0.001	0.42	0.20–0.92	0.03
Total mean volume (MET-hours)	0.76	0.70–0.81	< 0.001	0.86	0.79–0.94	0.001
Duration (Mean hours /day)	**Model 1**			**Model 2** ^b^		
Sedentary	1.54	1.29–1.84	< 0.001	1.14*	0.93–1.39	0.22
Light	0.93	0.72–1.21	0.59	1.26*	0.96–1.66	0.10
Moderate	0.30	0.20–0.46	<0.001	0.26^#^	0.14–0.47	<0.001
Vigorous	<0.001	<0.001–0.05	<0.01	0.01^#^	<0.001–0.86	0.04
Moderate to vigorous	0.31	0.21–0.47	< 0.001	0.52^#^	0.34–0.80	< 0.01
< 30 min /day	1.00			1.00		
30–60 min /day	0.28	0.08–0.97	0.04	0.30^#^	0.08–1.05	0.06
≥ 60 min /day	0.04	0.01–0.12	<0.001	0.10^#^	0.01–0.21	<0.001

Models 1 are adjusted for age, sex, education and smoking

Models 2^a^ are additionally adjusted for body mass index

Models 2^b^ are additionally adjusted for duration of moderate to vigorous* PA or duration of sedentary time^#^.

## Discussion

In this cross sectional study, our aim was to describe objectively measured daily PA among young Finnish adults, and further to examine associations between PA and MS, components of MS and body composition. We found that increasing total mean intensity (MET) and volume (MET-hours) of PA, were both associated with a reduced likelihood of having the MS. Longer duration of PA spent on sedentary level (<1.5 MET) was associated with an increased likelihood of having the MS and longer duration spent at moderate to vigorous level (≥ 3 MET) was associated with a decreased likelihood of having the MS. Higher mean volume of PA was inversely associated with body anthropometrics and body composition including BMI, waist circumference and body fat percentage. Our results are in line with previous literature suggesting beneficial effects of increasing PA levels or on the other hand, decreasing duration of sedentary behavior on cardio-metabolic health [16, 19, 31].

Although benefits of regular PA for health, morbidity and mortality are indisputable, it is still unclear, which component or pattern of PA is the key factor from a public health promotion aspect. Moreover total PA is highly dependent on age, lifestyle, general health and physical capacity, functioning and competence. PA or exercise performed at higher intensity levels may be required by some trained people to gain additional health benefits, while people with lower level of fitness may obtain health benefits from exercises performed even at a lower intensity levels. There are also divergent suggestions on whether the most effective health benefits are attained by sporadic or bouted PA. According to a recently published study, every additional weekly MET-hour decreased the odds of MS, regardless of whether PA of at least moderate level was accumulated in bouts or by sporadic activity [32].

It is not known whether the most important PA-induced health benefits are gained due to weight loss and diminished visceral fat accumulation or increased muscle mass and strength, or the combination of these. Adipose tissue is a complex, active metabolic and endocrine organ. Especially those with central obesity have accumulated fat in the subcutaneous abdominal and visceral depots and are prone to metabolic and cardiovascular complications [33]. A Finnish study conducted in young men during their military service showed that increased PA during the 8-week basic training period at the beginning of military service was the most effective means of health promotion for those who were obese and had MS. The estimated amount of PA correspond approximately 4 hours sports-related exercise training (such as running and strength training) and 12 hours of military-related physical training (such as marching and combat training). The prevalence of MS decreased in these young men mainly due to beneficial exercise-induced weight loss and changes in anthropometry, mainly in abdominal fat [34]. Findings from studies focusing upon the effects of PA, muscle strength or physical fitness on metabolic risk factors independently of body fat have been inconclusive [35–38]. It has been suggested that increasing muscular strength promotes health through metabolic and structural changes that improve muscle insulin sensitivity and glycemic control [39, 40].

Based on the results of an extensive study, approximately one third of adults worldwide, ranging from 17% to 43% depending on the continent and country, reported to be inactive and the proportion of youngsters aged 13 to 15 years doing less than 60 minutes of moderate to vigorous PA per day was as high as 80% [41]. In contrast to these proportions of self-reported activity, results from a study conducted in Sweden showed that objectively measured PA yielded even lower values and divergent activity pattern compared to those obtained by commonly used self-reports [42]. Compared to this literature, the young adults of our study are quite active; 90% being at least moderately active for over 60 minutes per day. Among them the prevalence of MS was 4%. For those who engaged in moderate to vigorous PA for 30–60 minutes per day, the prevalence of MS was 25% and 50% for those engaging less than 30 minutes.

Although an extensive literature exists on PA-related health benefits, it is challenging to make comparisons between different studies. Measuring PA at a population level is difficult because of the existence of a variety of methods for assessing PA in free living and field settings, including self-report questionnaires and different kinds of motion sensors each having their own specific features, advantages and disadvantages. Also there is no agreement on age- and gender- specific cut-off points for intensity levels. In the present study, no knowledge of participants’ maximum oxygen consumption level was available, which may have resulted in an over-estimation of physical activity level for some individuals from a physiological perspective.

Our study presents a sample of young adults with objectively measured PA. More-over body composition and anthropometrics were measured in a clinical setting with a validated BIA method suitable for analyses in an epidemiological setting. Both men and women were included in the study. With the use of the SenseWear Armband, a valid activity monitor, we were able to assess activity continuously for several days and objectively quantify a wide range of intensities.

However, there are some limitations in our study. The observations are cross-sectional, and therefore the detected associations do not indicate causality. Even though several confounding factors were controlled for, some other factors such as dietary habits and genetic factors which may have an impact on the results could not be taken into account. In addition, the current analysis does not include information about behavioral aspects of physical activity, such as type or mode, which could affect the observed associations. Furthermore there are suggestions that SenseWear Armband is not the most accurate in converting data of highest intensity activities, e.g. above 10 METs [26]. In conclusion, 8–10% of the study participants had the MS already at the age of 24 years. Our results suggest that increasing intensity and volume of PA and decreasing time spent at sedentary level of PA reduces the likelihood of developing the MS. The relatively high rate of MS found among young adults would support the early life prevention of metabolic and lifestyle diseases. According to our observations from the present data, we would call for more accurate age and gender specific thresholds when assessing intensity levels of PA.
